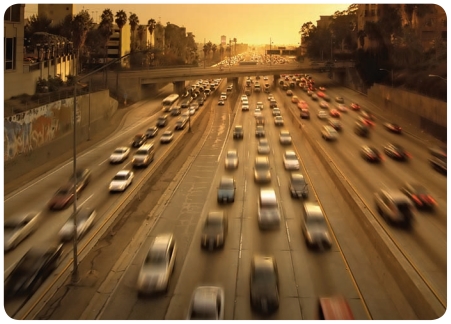# CARDIOVASCULAR HEALTH: Traffic Trigger

**DOI:** 10.1289/ehp.119-a205

**Published:** 2011-05

**Authors:** Wendee Holtcamp

**Affiliations:** **Wendee Holtcamp** writes about science and the environment from her home in Houston, TX. Her work has appeared in *Scientific American*, *Climate Central*, *Smithsonian*, and other publications

Do traffic-related stress and air pollution trigger more heart attacks than cocaine, caffeine, anger, or sex? New findings by a group of European researchers published in the 26 February 2011 issue of *The Lancet* suggests they may.^1^

When studying heart attack, or myocardial infarction, scientists look at two things: who has heart attacks and when. Many risk factors contributing to heart attacks are well known—hardening of the arteries (atherosclerosis), the chief factor underlying myocardial infarction, is strongly linked to cholesterol, diabetes, blood pressure, smoking, and family history of the condition. Within an at-risk individual, certain trigger events may set off a heart attack. Most research on heart attack triggers has focused on risk at an individual level, but this study took a novel approach by assessing the populationwide impacts of 13 different triggers.

The authors used a comparative risk assessment analysis using 36 previously published studies of nonfatal heart attack to determine the relative risk of each of 13 suspected triggers to both individual and overall public health. “One of the contributions of our paper, besides ranking the trigger factors, is that this is really an exercise in demonstrating the discrepancies between individual and population-based risks,” says lead author Tim Nawrot, an associate professor of environmental epidemiology at Hasselt University in Belgium.

The authors report that although certain triggers—including particulate air pollution and participation in traffic—were associated with relatively low risk at an individual level, on a population level they were associated with more total heart attacks. The triggers associated with heart attack at the individual level, ranked from highest to lowest risk, were cocaine use, a heavy meal, marijuana use, negative emotions, physical exertion, positive emotions, sexual activity/anger^2^/alcohol (tied), traffic exposure, respiratory infection, coffee consumption, and air pollution. But when the authors looked at the population attributable fraction (PAF)—or the proportion of heart attacks preceded by each trigger—the ranking changed. Traffic exposure (potentially including both air pollutant and stress exposures) was associated with more heart attacks than any other single factor (see table).

Population-level rankings are determined by the prevalence of exposure, as well as the level of risk associated with the exposure. “Many more people are exposed to increased levels of air pollutants more frequently than to many other triggering risk factors, so from a population standpoint, air pollution may be more important,” says Joel Kaufman, a professor of environmental and occupational medicine at the University of Washington. He points out that air pollution exposure is not something people can always make a choice about, unlike smoking or cocaine use. Other known triggers such as temperature extremes^3^ and secondhand tobacco smoke^4^ were not addressed in the study, nor were interactions among triggers.

Kaufman said he might quibble with some numbers in the paper. “It’s not clear that [the authors] used consistent or realistic methods to determine the prevalence of exposure for air pollution versus other things,” he says. “For air pollution they’ve been rather generous by listing it at a hundred percent while some other factors were given what seem to be surprisingly low or high assignments of exposure prevalence that don’t reflect the general population at risk for heart attacks.” But he says this does not change the study’s message.

Epidemiology professor Charles Poole of the University of North Carolina at Chapel Hill has concerns over using PAFs in studies of public health impacts. Essentially, PAFs assume it is possible to completely eliminate a trigger, which is highly unlikely for things like emotions or sexual activity. “[PAFs] are vast overstatements of the amount by which heart attack incidence could be reduced,” Poole says. “It’s proposed as a way of guiding public health measures and policy decisions [that] is grossly unrealistic.”

On the other hand, Poole liked that Nawrot et al. used realistic reductions in particulate air pollution of 10 and 30 μg/m^3^ rather than complete elimination. He says that, although triggers are worthy of study, from a public health perspective it is more important to focus on reducing the number of people who are one trigger event away from a heart attack.

An expert panel of the American Heart Association issued a statement^5^ in 2010 (updating their 2004 statement^6^) indicating that a substantial amount of evidence has accumulated suggesting air pollution triggers heart attacks; what’s more, they said plausible mechanisms exist for how air pollution may contribute to coronary atherosclerosis. In other words, exposure to air pollution also puts more people at risk for developing heart disease. In that sense, air pollution is a public health concern that both scientists and practitioners are starting to take seriously.

**Proportion of heart attacks preceded by each of 13 trigger events: population level^1^**

7.4^%^ **traffic exposure**

6.2^%^ **physical exertion**

5.0^%^ **alcohol**

5.0^%^ **coffee**

3.9^%^ **negative emotions**

3.1^%^ **anger**

2.7^%^ **heavy meal**

2.4^%^ **positive emotions**

2.2^%^ **sexual activity**

0.9^%^ **cocaine use**

0.8^%^ **marijuana use**

0.6^%^ **respiratory infection**

## Figures and Tables

**Figure:**